# Association between Optic Nerve Sheath Diameter and Lamina Cribrosa Morphology in Normal-Tension Glaucoma

**DOI:** 10.3390/jcm12010360

**Published:** 2023-01-02

**Authors:** Seung Hyen Lee, Tae-Woo Kim, Eun Ji Lee, Hyunkyung Kil

**Affiliations:** 1 Department of Ophthalmology, Nowon Eulji Medical Center, Eulji University College of Medicine, Seoul 01830, Republic of Korea; 2 Department of Ophthalmology, Seoul National University Bundang Hospital, Seoul National University College of Medicine, Seongnam 13620, Republic of Korea; 3 Department of Ophthalmology, Bundang Jesaeng General Hospital, Seongnam 13590, Republic of Korea

**Keywords:** optic nerve sheath diameter, normal-tension glaucoma, lamina cribrosa curvature

## Abstract

(1) Background: To compare optic nerve sheath diameter (ONSD) in normal-tension glaucoma (NTG) and healthy eyes and to investigate the association between ONSD and lamina cribrosa (LC) morphology. (2) Methods: This cross-sectional study included 69 NTG eyes and 69 healthy eyes matched for age, axial length, and intraocular pressure. The LC curvature index (LCCI) was measured from horizontal Cirrus HD-OCT B-scan images from five uniformly divided positions vertically of the optic nerve. The average LCCI was defined as the mean of the measurements at these five locations. ONSD was measured as the width of the optic nerve sheath at the site perpendicular 3 mm behind the posterior globe. LCCI and ONSD were compared in eyes with NTG and healthy eyes. The clinical factors that could affect LCCI were analyzed. (3) Results: NTG eyes had significantly smaller mean ONSD (4.55 ± 0.69 mm vs. 4.97 ± 0.58 mm, *p* < 0.001) and larger average LCCI (11.61 ± 1.43 vs. 7.58 ± 0.90, *p* < 0.001) than matched healthy control eyes. LCCI was significantly correlated with smaller ONSD, higher intraocular pressure, thinner global retinal nerve fiber thickness, and worse visual field loss in all subjects (all Ps ≤ 0.022). (4) Conclusions: NTG eyes had smaller ONSD and greater LCCI than healthy control eyes. In addition, a negative correlation was observed between ONSD and LCCI. These findings suggest that cerebrospinal fluid pressure, which ONSD indirectly predicts, may affect LC configuration. Changes in the retrolaminar compartment may play a role in glaucoma pathogenesis.

## 1. Introduction

Glaucoma is characterized by progressive optic neuropathy and equivalent visual field defects. In the pathophysiology of glaucoma, the most crucial independent risk factor is well known as the mechanical stress associated with an increase in intraocular pressure (IOP) [1,2]. However, glaucomatous optic nerve damage can develop and progress even when the IOP is in the normal range, a condition called normal-tension glaucoma (NTG). The pathophysiology of NTG, however, has not been fully determined.

The lamina cribrosa (LC) structurally supports the pathway of retinal ganglion cell axons and divides the optic nerve into two parts: the intraocular space anteriorly and the retrobulbar space posteriorly [3]. Experimental and clinical studies have shown that the LC morphology changes depending on the magnitude of IOP [4,5]. In an experimental model of glaucoma, it has been found that an IOP elevation induced the LC to curve posteriorly [4]. In contrast, the LC became less bowed when the IOP was lowered by IOP reduction treatment [5]. In addition to the effect of IOP on the LC, increase of cerebrospinal fluid (CSF) pressure also raised the strain of LC and retrolaminar neural tissue [6,7]. These findings indicate that the translaminar pressure gradient (TLPG) between the intraocular and retrobulbar space/tissue can induce LC deformation. Interestingly, the greater degree of backward LC bowing observed in eyes with high tension glaucoma, relative to healthy eyes, was also observed in eyes with NTG [8,9]. This finding suggests that TLPG may be still greater in eyes with NTG than in healthy eyes.

Since the optic nerve is surrounded by CSF, CSF pressure is closely related to retrolaminar tissue pressure [10]. However, invasive methods are required to measure CSF pressure. An alternative noninvasive approach, measurement of optic nerve sheath diameter (ONSD) by ultrasonography, is proposed as a surrogate parameter to estimate CSF pressure [11,12]. ONSD was found to be smaller in NTG eyes than in healthy control eyes in several previous studies, suggesting that CSF pressure is likely lower in NTG than in healthy subjects [13,14]. This finding gives an explanation that TLPG may be high in eyes with NTG, despite IOP being within the normal range. We hypothesized that, if low CSF pressure plays a significant role in generating high TLPG in NTG, thereby promoting posterior deformation of the LC, there would be a significant relationship between ONSD and the degree of backward LC bowing.

The purpose of this study was to compare ONSD in eyes with NTG and healthy control eyes and to investigate the relationship between LC morphology and ONSD in eyes with NTG.

## 2. Materials and Methods

The electronic medical records of all subjects who visited Bundang Jesaeng Hospital Glaucoma Clinic between February 2017 and December 2020 were retrospectively reviewed. The IRB of Bundang Jesaeng Hospital approved this study, which was conducted in accordance with the Helsinki Declaration.

### 2.1. Study Subjects

All subjects received general ophthalmic examinations as described elsewhere [15]. Stereo disc photography with a CR-2 Plus AF (Canon Inc., Tokyo, Japan), Cirrus HD-OCT (Carl Zeiss-Meditec, Jena, Germany), immersion B-scan ultrasonography (Quantel Medical compact II; Quantel Medical), central corneal thickness (Orbscan II), axial length (IOL Master 500; Carl Zeiss Meditec Ltd, Jena, Germany) and 24-2 Humphrey perimetry (Carl Zeiss Meditec, Jena, Germany) were also performed.

Eyes included in this study needed to have a best-corrected visual acuity 20/40 or better, −6.0D or higher and +3.0D or lower spherical refraction, –3.0 to +3.0 D for cylinder correction, normally open anterior chamber angle, and reliable visual field tests. The following eyes were excluded; (1) optic nerve tilt [16,17] or torsion [17,18] to remove the possibility that the LC may be deformed by other reasons, (2) intraocular surgery history excluding cataract operation, (3) diagnosis other than normal-tension glaucoma (e.g., secondary glaucoma), (4) neurologic disorders that can affect visual field loss, (5) low-quality image (i.e., signal strength < 7) caused by media opacity or patient incorporation, (6) ill-defined anterior surface of the central LC.

NTG was defined as having of glaucomatous optic neuropathy with a corresponding glaucomatous visual field defect; the maximum IOP without medications ≤21 mmHg; open angle on gonioscopy, and no secondary cause of glaucoma. A glaucomatous visual field defect was confirmed in two consecutive tests as described elsewhere [15].

The healthy control subjects were defined as eyes with normal IOP (≤21 mmHg) without an increase in IOP, no glaucomatous optic disc, no obvious retinal nerve fiber layer (RNFL) defects, a circumpapillary RNFL thickness measured by OCT in the normal range (within the 95th percentile of the normal data) and a normal visual field results (without glaucomatous or neurologic field defects).

One eye was randomly selected when both eyes met the inclusion criteria. Age, axial length, and IOP were matched between NTG and healthy control eyes using a frequency matching method.

Baseline IOP was defined as the average of the values measured 5 or more times prior to the initiation of IOP-lowering therapy and scan IOP was measured on the day of taking Cirrus HD-OCT images.

### 2.2. Cirrus High-Definition Optical Coherence Tomography Imaging of the Optic Nerve Head

Images of the optic nerve head of all subjects was obtained by a single well-trained technician using the Cirrus HD OCT 6000. Subjects with high-quality scans were included for analysis. High-quality OCT images were defined as those with a signal strength ≥ 7 (maximum, 10) without any motion artifacts, involuntary saccades, apparent decentration misalignment, or algorithm segmentation error. Optic disc images were obtained using an optic disc cube protocol of a 6 × 6 mm^2^ area composed of 40,000 points (200 × 200 axial scans) and 21-HD line raster scans (9 mm length in enhanced depth imaging [EDI] mode). The built-in analysis algorithm (software version 11.5; Carl Zeiss Meditec, Jena, Germany) detects the center of the optic disc and calculates the peripapillary RNFL thickness at the 3.46 mm diameter circle automatically from the dataset consisting of 256 A-scans.

### 2.3. Lamina Cribrosa Curvature Index Measurement

**Figure 1:**
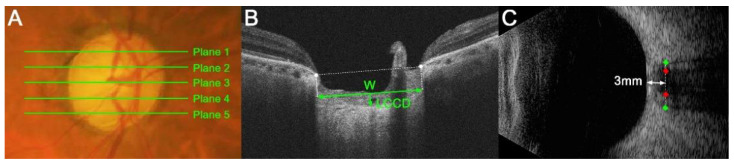
Measurements of the lamina cribrosa (LC) curvature index (LCCI), optic nerve sheath diameter (ONSD) and optic nerve diameter (OND). (**A**) Stereoscopic optic disc photograph image. The five horizontal green lines indicate the locations where the measurements were performed. (**B**) Cirrus HD OCT B-scan images were obtained at plane 4 as shown in (**A**). The LCCI was measured by dividing the LC curve depth (LCCD) by the length of the LC surface reference line (W) within Bruch’s membrane opening (BMO) and multiplying by 100. (**C**) Transorbital ultrasonographic image. The ONSD (distance between green dots) and OND (red dots) were assessed perpendicular to the scanning place 3 mm behind the globe.

LC morphology was quantified by a measurement of the LC curvature index (LCCI). The LCCI was determined how curved the LC is, as described previously [8,15]. Briefly, the line connecting two points that descend vertically from the two Bruch’s membrane opening points until the anterior border of the LC was defined as the LC surface line. Lamina cribrosa curve depth (LCCD) was the maximal depth from LC surface line to LC surface and the width (W) was defined as the length of this LC surface line. The LCCI was estimated as followed: LCCI = (LCCD/W) × 100 (Figure 1B) [19].

The measurement of LCCIs was performed at five locations dividing the optic nerve into 5 equal parts vertically using the built-in caliper of the viewer program (software version 11.5; Carl Zeiss Meditec, Jena, Germany) twice by a single experienced observer (SHL) blinded to the clinical information. The analysis used average of the two values measured twice. The average LCCI was defined as the mean values of measurements at the five locations.

### 2.4. Measurement of Optic Nerve Sheath Diameter and Optic Nerve Diameter

B-scan images were acquired by a single experienced technician who was blinded to the study protocol using transorbital ultrasonography with a linear 10-MHz probe. The patient lied in a supine position, followed by placement of sterile gel and probing slightly without pressure on the eyelid to prevent eye damage. The transverse view of the globe and retrobulbar part was obtained by holding the probe with the axial plane on the eyelid.

The ONSD and OND were defined as the optic nerve sheath width and the optic nerve width perpendicular to the vertical axis of the scanning site 3 mm behind the globe (Figure 1C), respectively. A position 3 mm behind the eyeball was a site that is susceptible to expansion due to rises in intracranial pressure (ICP) [20,21]. It was also the site of the greatest ultrasound contrast and the optimal distensibility of the optic nerve sheath in terms of CSF dynamics [20]. The ONSD and OND in all eyes were measured twice by a single glaucoma specialist (LSH) using Image J software (version 1.50 e; National Institutes of Health, Bethesda, MD, USA) in a masked manner, with the average of the two measured values used for the analysis.

### 2.5. Statistical Analysis

The intra-observer agreement between LCCI, ONSD and OND measurements was assessed using Bland-Altman limits of agreement and intra-class correlation coefficients. Independent *t*-tests (for continuous variables) and chi-square tests (for categorical variables) were performed in the comparison between the two groups. Bonferroni’s correction was applied to *t*-tests based on the number of locations. Linear regression analyses were conducted to evaluate the factors related to LCCI. Fisher’s z transformation was used to evaluate difference in the correlation coefficient between groups. *p* values < 0.05 were considered as statistically significant. The Statistical Package for Social Sciences was used in all statistical analyses (ver. 22.0, SPSS, Inc. Chicago, IL, USA).

## 3. Results

### 3.1. Demographic and Clinical Characteristics

This retrospective study initially involved 117 healthy control eyes and 98 eyes with NTG. Of these eyes, eight healthy control and 19 NTG eyes were excluded because of poor quality of OCT images, in which the anterior LC contour cannot be clearly distinguished even in one of five images of disc scan. In addition, two healthy control and five NTG eyes were excluded due to poor ultrasonographic image quality that did not allow a clear delineation of the optic nerve sheath margin. After matching the groups for age, IOP and axial length, 69 eyes of 69 NTG patients and 69 eyes of 69 healthy control subjects were finally included.

Table 1 presents the demographic characteristics of the study subjects. There were significant differences in mean deviation and pattern standard deviation on visual field tests, global RNFL thickness and ONSD (all *p* ≤ 0.002, Table 1). ONSD was significantly lower in eyes with NTG than in healthy control eyes (4.55 ± 0.69 mm vs. 4.97 ± 0.58 mm, *p* < 0.001). The 95% Bland-Altman limits of agreement were −1.12 mm to 1.07 mm, −0.74 mm to 0.61 mm and −1.49 to 1.62, for the measurements of ONSD, OND and LCCI, respectively. The intra-class correlation coefficients for the interobserver reproducibility in measuring the ONSD, OND, and LCCI were 0.975, 0.987, and 0.967, respectively.

### 3.2. Comparison of LCCI in the Two Groups

Table 2 compares LCCI in NTG and healthy control eyes. The average LCCI in NTG eyes was 11.61 ± 1.43. In all five planes, the LCCI was significantly larger in NTG than in healthy control eyes (all *p* < 0.001).

### 3.3. Factors Associated with LCCIs

**Figure 2:**
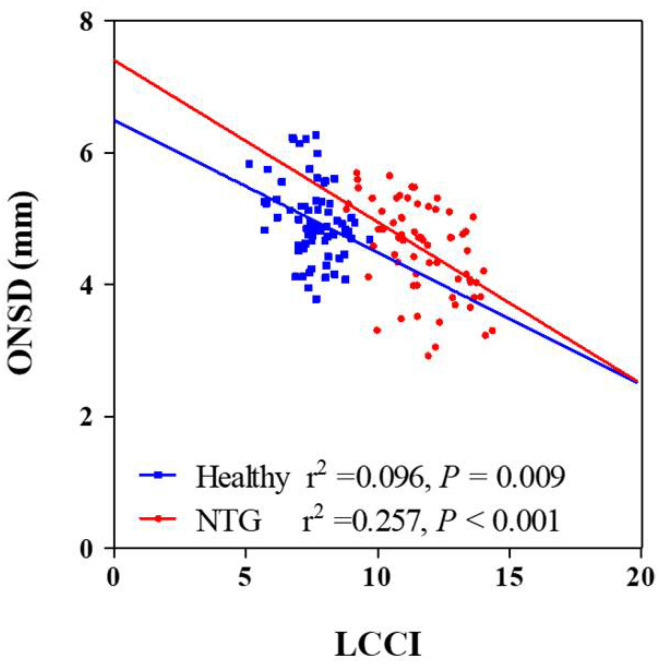
Scatterplots showing the relationship between lamina cribrosa curvature index and clinical parameters. Solid lines represent trend lines.

Univariate analysis showed that higher scan IOP, thinner global RNFL thickness, worse visual field loss and smaller ONSD were significantly associated with average LCCI (all Ps ≤ 0.019. In order to exclude multicollinearity between global RNFL thickness and visual field MD (variance inflation factor [VIF] = 4.000 and 3.972, respectively) and obtain reliable results, multivariate analysis was performed using two models. The average LCCI showed significant negative association with ONSD, global RNFL thickness, and visual field loss (all Ps < 0.001, Table 3) and significant positive correlation with scan IOP (Ps ≤ 0.022) in all subjects (Table 3; Figure 2) in both models. There was no significant difference of correlation coefficient between NTG and healthy groups (z score = 1.36, *p* = 0.087).

### 3.4. Representative Subjects

**Figure 3:**
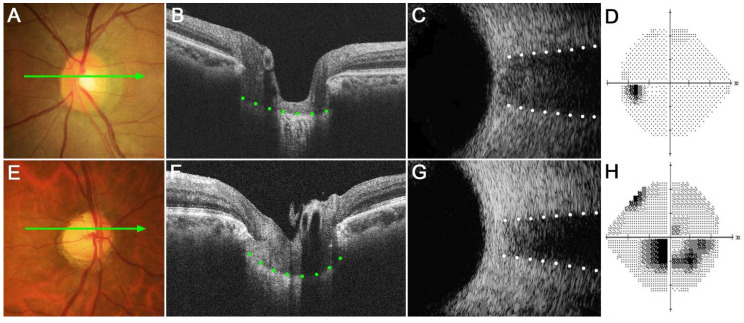
Representative eyes showing the relationship between the lamina cribrosa curvature index (LCCI) and optic nerve sheath diameter (ONSD). (**A**–**D**) A healthy left eye of a 63-year-old woman with a relatively flat LC and wide ONSD. (**E**–**H**) A glaucomatous right eye of a 59-year-old man with curved LC and narrow ONSD. (**A**,**E**) Stereoscopic optic disc photograph images. (**B**,**F**) Cirrus HD OCT B-scan images obtained at the locations indicated by light green arrows in (**A**,**E**), respectively. (**C**,**G**) Transorbital ultrasound images showing that ONSD was larger in the healthy eye (**C**) than in the glaucomatous eye (**G**). Note that LCCI is considerably larger in (**F**) than in (**B**), whereas the ONSD is notably larger in (**C**) than in (**G**). The comparison of these two representative eyes indicates a negative correlation between LCCI and ONSD.

Figure 3 shows two eyes, a healthy control eye (Figure 3A–D) and a glaucomatous eye (Figure 3E–H). The healthy eye had a relatively flat LC with wide ONSD, whereas the glaucomatous eye had a curved LC with a narrow ONSD. Note that LCCI was larger and ONSD was smaller in the NTG than in the healthy eye.

## 4. Discussion

The present findings demonstrated that NTG eyes have smaller ONSD and larger LCCI than healthy control eyes, with a significant negative correlation between ONSD and LCCI. To our knowledge, this study is the first to investigate the relationship between ONSD and LC morphology.

In accordance with previous findings, the present study found that average LCCI was significantly larger in eyes with NTG than in healthy control eyes [15]. This finding suggests that TLPG is still relatively high in NTG eyes despite IOP being within the normal range.

Methods that measure CSF pressure, such as lumbar puncture, are limited in clinical practice because of their invasiveness. In contrast, ultrasound-based assessment of ONSD is a noninvasive method used clinically to detect increases in ICP in emergency and intensive care units [11,22,23]. The subarachnoid space surrounding the optic nerve does not directly communicate [10,24], but still connected with the rest of the CSF space [25]. Therefore, CSF pressure has been shown to generally correlate with ONSD in several previous studies [11,12,26]. Since the optic nerve sheath is elastic, ONSD may increase when the CSF pressure is increased [27]. Conversely, ONSD is reduced in patients with intracranial hypotension [28,29]. Therefore, measurements of ONSD have been used to investigate the role of CSF pressure in the pathophysiology of glaucoma [13,14,30,31].

The present study found that ONSD was significantly smaller in NTG eyes than in healthy control eyes matched for age, IOP and axial length. Given that ONSD indirectly represents CSF pressure, these results suggest that CSF pressure is lower in NTG than in healthy eyes. Until now, it is inconclusive whether CSF pressure is high or low in the NTG [32,33]. These present findings are consistent with previous experimental and clinical studies showing that CSF pressure is abnormally decreased in NTG [33,34,35,36]. These observations are also in agreement with previous studies that the subarachnoid space is narrower in NTG eyes than in high-tension glaucoma and healthy control eyes [13,14]. Taken together, these findings indicate that low CSF pressure plays a significant role in NTG.

The response of the LC according to the TLPG would be inevitably affected by the material properties of the LC. Until now, LC properties such as rigidity cannot be directly measured. It is generally known that the LC stiffens with age [37], and the LC becomes thinner as the axial length increases [38]. We tried to minimize the effect of these LC properties on LC morphology by matching age and axial length between two groups.

**Figure 4:**
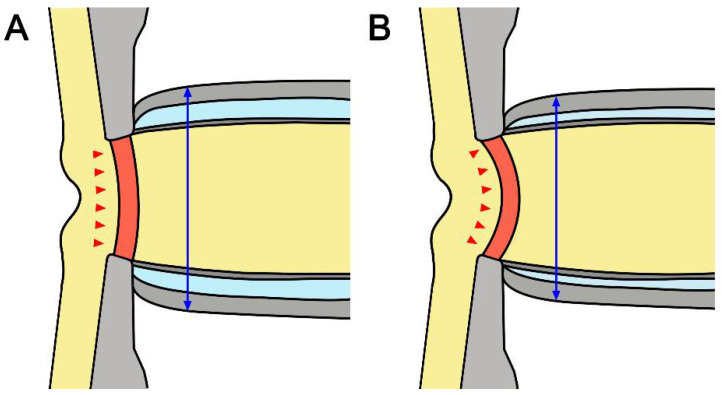
Schematic diagram of the optic nerve in healthy (**A**) and glaucomatous (**B**) eyes. Note that smaller ONSD and more posteriorly curved LC in NTG suggested that low cerebrospinal fluid pressure may induce a relatively higher TLPG in NTG than in healthy eyes. The double headed blue arrows indicate ONSD and the red arrows represent the morphology of LC.

It is noteworthy that there was a negative correlation between average LCCI and ONSD in all subjects. LC curvature may be determined by the net effect of the TLPG and material properties. Therefore, not only IOP but also retrolaminar tissue pressure may contribute to the configuration of the LC. Although the material properties of the LC are also related, the correlation between LCCI and ONSD in NTG eyes suggests that low CSF pressure is related to the greater LCCI in NTG than in healthy eyes (Figure 4).

The average ONSDs measured in healthy control (4.97 ± 0.58 mm) and NTG (4.55 ± 0.69 mm) eyes in the present study are comparable to those previously reported in studies that measured ONSD using ultrasound [13,39] and magnetic resonance imaging [14]. In addition, a recent meta-analysis found that ONSD cut-off values for assessing intracranial hypertension varied from 4.80 to 6.30 mm [40].

The present study had limitations. First, this study did not consider several parameters potentially associated with the response of the LC, such as LC thickness and the material properties of the laminar and peripapillary scleral connective tissue [41]. These parameters, however, are difficult or impossible to measure currently. As new technology advances make these parameters measurable, a more reliable relationship between LC morphology and postlaminar compartment will be obtained. Second, actual measurement of CSF pressure was not performed in the present study, then it could not be definitely concluded that the smaller ONSD would be correlated with lower CSF pressure. However, the validity of using ONSD as a surrogate to CSF pressure has been suggested in multiple studies [12,42,43]. Third, a tilted or torted optic disc were not included, therefore our results cannot be applicable to these eyes directly. Forth, LCCI measurements using OCT were performed in the sitting position, and ONSD measurements using ultrasonography were made in the supine position. Since CSF pressure may vary depending on body posture, it is possible that the result was affect by a difference in body position. However, modalities of the two measurement methods were unified in each setting, it would not have had a significant effect on the results.

In conclusion, ONSD was smaller and LCCI was larger in NTG than in healthy control eyes. These findings are consistent with the concept that lower CSF pressure may play a significant role in the pathogenesis of glaucomatous optic neuropathy in NTG.

## Figures and Tables

**Table 1:** Demographic characteristics of the study subjects.

Variables	Healthy Control Eyes (n = 69)	NTG Eyes (n = 69)	*p* Value
Demographic characteristics			
Age, years	67.8 ± 10.2	67.8 ± 9.8	0.986
Female, n (%)	33 (47.8)	36 (52.2)	0.734
Diabetes mellitus, n (%)	17 (24.6)	18 (26.1)	0.845
Hypertension, n (%)	35 (50.7)	34 (49.3)	0.865
Ophthalmic characteristics			
Baseline IOP, mmHg	16.9 ± 2.1	n/a	
Scan IOP, mmHg	14.9 ± 2.7	14.8 ± 2.5	0.718
Spherical error, diopters	−0.41 ± 2.25	−1.11 ± 2.86	0.111
Axial length, mm	23.75 ± 0.96	23.86 ± 1.18	0.543
Central corneal thickness, μm	547.1 ± 37.4	534.7 ± 29.0	0.124
VF MD, dB	−1.18 ± 2.30	−10.59 ± 8.24	**<0.001**
VF PSD, dB	1.59 ± 2.80	6.71 ± 3.76	**0.002**
Global RNFL thickness, μm	88.0 ± 8.9	68.2 ± 12.7	**<0.001**
ONSD, mm	4.97 ± 0.58	4.55 ± 0.69	**<0.001**
OND, mm	3.29 ± 0.35	3.21 ± 0.36	0.174

NTG = normal tension glaucoma; IOP = intraocular pressure; VF = visual field; MD = mean deviation; dB = decibel; PSD = pattern standard deviation; RNFL = retinal nerve fiber layer; ONSD = optic nerve sheath diameter; OND = optic nerve diameter. Data are reported as mean ± standard deviation or as number (percent), with statistically significant *p* values in boldface.

**Table 2:** Comparison of LCCI in healthy and NTG eyes.

	Healthy Eyes (n = 69)	NTG Eyes (n = 69)	*p* Value
Plane 1	7.88 ± 1.09	12.07 ± 1.78	**<0.001**
Plane 2	7.71 ± 1.28	11.77 ± 1.77	**<0.001**
Plane 3	6.89 ± 1.29	10.55 ± 1.68	**<0.001**
Plane 4	7.60 ± 1.38	11.34 ± 2.00	**<0.001**
Plane 5	7.85 ± 1.25	12.08 ± 2.06	**<0.001**
Average	7.58 ± 0.90	11.61 ± 1.43	**<0.001**

LCCI = lamina cribrosa curvature index; NTG = normal-tension glaucoma. Data are mean ± standard deviation values. Bonferroni correction was applied to raw data for measurements in the five planes. Values significant after Bonferroni correction (*p* < 0.01; 0.05/5) are shown in bold.

**Table 3:** Factors associated with LCCI in all subjects.

	All Subjects (n = 138)							
	Univariate		Multivariate Model 1			Multivariate Model 2		
Variables	Beta	*p*	Beta	*p*	VIF	Beta	*p*	VIF
Age, per 1-year older	0.008	0.706						
Gender, female	−0.299	0.455						
Diabetes mellitus	0.230	0.617						
Hypertension	−0.207	0.606						
Scan IOP, mmHg	0.182	**0.019**	0.129	**0.022**	1.019	0.151	**0.012**	1.019
Spherical equivalent, diopter	−0.142	0.166						
Axial length, mm	0.127	0.498						
Central cornea thickness, μm	−0.004	0.663						
Global RNFL thickness, μm	−0.096	**<0.001**	−0.083	**<0.001**	1.059			
Visual field MD, dB	−0.169	**<0.001**				−0.147	**<0.001**	1.052
ONSD, mm	−1.666	**<0.001**	−1.168	**<0.001**	1.078	−1.253	**<0.001**	1.066

IOP = intraocular pressure; RNFL = retinal nerve fiber layer; MD = mean deviation; dB = decibel; ONSD = optic nerve sheath diameter. Only variables with *p* < 0.1 on univariate analysis were included in the multivariate model. Statistically significant factors are shown in boldface.

## Data Availability

The data presented in this study are available on request from the corresponding author. The data are not publicly available due to privacy issues.
